# Is social media, as a main source of information on COVID-19, associated with perceived effectiveness of face mask use? Findings from six sub-Saharan African countries

**DOI:** 10.1177/17579759211065489

**Published:** 2022-01-27

**Authors:** Ihoghosa Iyamu, Glory Apantaku, Zeena Yesufu, Edward Adekola Oladele, Ejemai Eboreime, Barinaadaa Afirima, Emeka Okechukwu, Gabriel Isaac Kibombwe, Tolulope Oladele, Taurayi Tafuma, Okiki-Olu Badejo, Everline Ashiono, Mulamuli Mpofu

**Affiliations:** 1 Pan African Research Consortium, Federal Capital Territory, Abuja, Nigeria; 2 School of Population and Public Health (SPPH), University of British Columbia, Vancouver, Canada; 3 Episolution Public Health Services, Federal Capital Territory, Abuja, Nigeria; 4 National Primary Health Care Development Agency (NPHCDA), Abuja, Nigeria; 5 Department of Psychiatry, Faculty of Medicine and Dentistry, University of Alberta, Edmonton, Canada; 6 FHI 360 (Family Health International), Tanzania; 7 FHI 360 (Family Health International), Zambia; 8 National Agency for the Control of AIDS (NACA), Abuja, Nigeria; 9 Department of Public Health, Institute of Tropical Medicine, Antwerp, Belgium; 10 Egerton University, Kenya

**Keywords:** nonpharmaceutical interventions, COVID-19, social media, face masks, health promotion

## Abstract

**Background:**

The use of face masks as a public health approach to limit the spread of coronavirus disease 2019 (COVID-19) has been the subject of debate. One major concern has been the spread of misinformation via social media channels about the implications of the use of face masks. We assessed the association between social media as the main COVID-19 information source and perceived effectiveness of face mask use.

**Methods:**

In this survey in six sub-Saharan African countries (Botswana, Kenya, Malawi, Nigeria, Zambia and Zimbabwe), respondents were asked how much they agreed that face masks are effective in limiting COVID-19. Responses were dichotomised as ‘agree’ and ‘does not agree’. Respondents also indicated their main information source including social media, television, newspapers, etc. We assessed perceived effectiveness of face masks, and used multivariable logistic models to estimate the association between social media use and perceived effectiveness of face mask use. Propensity score (PS) matched analysis was used to assess the robustness of the main study findings.

**Results:**

Among 1988 respondents, 1169 (58.8%) used social media as their main source of information, while 1689 (85.0%) agreed that face masks were effective against COVID-19. In crude analysis, respondents who used social media were more likely to agree that face masks were effective compared with those who did not [odds ratio (OR) 1.29, 95% confidence interval (CI): 1.01–1.65]. This association remained significant when adjusted for age, sex, country, level of education, confidence in government response, attitude towards COVID-19 and alternative main sources of information on COVID-19 (OR 1.33, 95%CI: 1.01–1.77). Findings were also similar in the PS-matched analysis.

**Conclusion:**

Social media remains a viable risk communication channel during the COVID-19 pandemic in sub-Saharan Africa. Despite concerns about misinformation, social media may be associated with favourable perception of the effectiveness of face masks.

## Introduction

The novel coronavirus disease 2019 (COVID-19) pandemic continues to pose significant challenges for health systems around the world (1). Despite the development of new vaccines, the emergence of new viral strains of concern, delays and logistic challenges inherent in large scale immunisation campaigns across countries of the world reinforce the need to strengthen existing nonpharmaceutical interventions (NPI) to limit disease spread (2,3). One such NPI that has gained public interest is the use of face masks by individuals in the community as a way to prevent disease spread, especially from infected persons who are asymptomatic (3–6). The World Health Organisation (WHO) and other health authorities in various jurisdictions have made evolving and sometimes confusing recommendations about this issue (6,7).

There is growing concern about the role of social media in spreading misinformation about the effectiveness of face masks and other NPIs in preventing the spread of COVID-19 (8,9). While concerns about health misinformation via social media are not new, the COVID-19 pandemic has amplified these concerns (9–12). Suboptimal regulation of information sources and the propensity for social media algorithms to prioritise the most popular posts make it inherently difficult for the public to verify health information via modern media channels like Twitter, Facebook and Instagram, and messaging platforms like WhatsApp (9,13,14). Yet these channels are major channels for risk communication and health promotion, especially in health emergencies like COVID-19 (10,11,15).

In resource-limited settings like sub-Saharan Africa, the importance of social media in health prevention and promotion, especially during COVID-19, cannot be overstated (16). However, social media has been seen as a medium for misinformation, especially about reduced vulnerability to COVID-19 and the availability of untested therapies (17,18). Concerted efforts at misinformation have been shown to be often politically motivated, especially in a health emergency like COVID-19, resulting in the development of an ‘infodemic’ – a situation defined by the uncontrolled spread of low-credibility, false, misleading and unverified information (11,12,17). Misinformation via social media is also suggested to be fuelling untoward perceptions of the effectiveness of NPIs, particularly the use of face masks (19–21). Despite these concerns, evidence is limited on the relationship between the use of social media as the main COVID-19 information source and perceived effectiveness of face masks as a public health strategy.

The limited and emerging evidence suggests that social media may play a role in informing people’s perception of the effectiveness of face mask use (22). Yet, no study has specifically assessed this relationship in the sub-Saharan African region. This region may have escaped the first wave and second waves of the COVID-19 with relatively less morbidity and mortality than the rest of the world, but emerging data from the third wave is raising concerns as morbidity and mortality rates are on the increase (23–25). More evidence is required to inform ongoing public health engagement strategies that will continue to protect the health of Africans in subsequent waves. In this context, this study seeks to assess the association between use of social media as the main COVID-19 information source and perceived effectiveness of face mask use in six sub-Saharan countries.

## Methods

### Study design, setting and population

We conducted a cross-sectional survey of 1198 respondents from six sub-Saharan African countries: Botswana, Kenya, Malawi, Nigeria, Zambia and Zimbabwe. These countries, although largely diverse, share similarities. In terms of the variations, population sizes range from 2.2 million in Botswana to about 200 million in Nigeria (26). However, there is a shared growth in the adoption of mobile and internet technologies that facilitate access to social media platforms. For example, between January 2019 and January 2020, the number of internet users increased by 2.2 million (2.6%), 3.2 million (16%) and 595,000 (16%) in Nigeria, Kenya and Zambia, respectively (27). Large variations in education have been noted for the selected countries. For instance, less than 1% of Zimbabwean children of primary school age are out of school. The same applies to Malawi, where only 2% of children are out of school (26). However, 15%, 19% and 34% of children were reported out of school in Zambia, Kenya and Nigeria, respectively (26).

### Sample size and sampling

We selected a sample of respondents from six countries in West (1), East/Central (1) and Southern Africa (4). These countries were selected to give a geographic representation across the different sub-Saharan African blocs that typically differ in national culture and context. For each country, since the population was greater than 20,000, we determined, at 95% confidence level, a sample of 384 respondents to have sufficient power to provide generalisable results in each country at a total sample size of 2304 (28).

### Data collection

The survey was administered online, between 17 May 2020 and 15 June 2020 using structured questionnaires on Google forms (Alphabet Inc., Mountain View, CA, USA), with appropriate skip logics and patterns as indicated. Respondents were recruited via email listservs, Facebook, Twitter, Telegram and WhatsApp. Enrolment in the study occurred on a first-come, first-served basis. As part of the survey, we assessed respondents’ perceived effectiveness of face mask use in limiting COVID-19, and their main source of information including social media, television, newspapers, employers, family, friends, and online/web channels. Further, data on respondents’ sociodemographic characteristics, COVID-19 risk perception and attitude to COVID-19 were collected.

### Analytic sample and study variables

**Figure 1.**
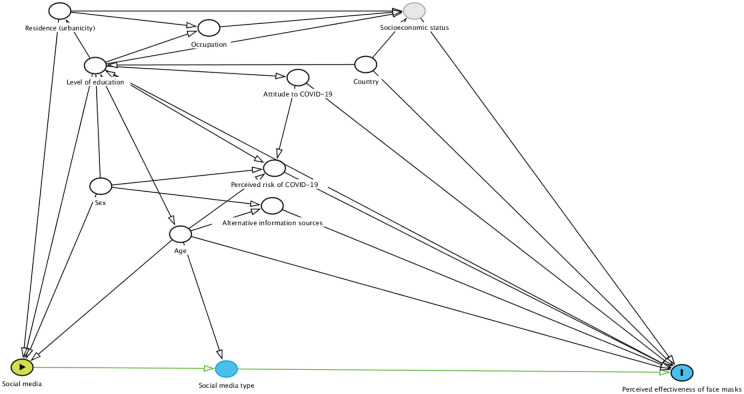
The DAG for assessing the relationship between social media as a main source of COVID-19 information and perceived effectiveness of face masks as an NPI for COVID-19. This illustrates the confounding effects of age, sex, occupation, country, level of education, confidence in government response, perceived COVID-19 risk, attitude towards COVID-19, first source of information on COVID-19, and alternative main sources of information on COVID-19 including television, radio, friends and family, online/websites, newspapers and employers. It also shows social media type as a mediator of this association. Socioeconomic status is an unmeasured variable. COVID-19: coronavirus disease 2019; DAG: directed acyclic graph; NPI: nonpharmaceutical intervention.

Our study sample included all respondents who had valid responses to our outcome question, which assessed how much they agreed that the use of face masks was effective in limiting COVID-19 in their countries, on a 5-point Likert scale ranging from ‘strongly disagree’ to ‘strongly agree’. Responses were dichotomised as ‘agree’ and ‘does not agree’. Responses such as ‘don’t know’, or ‘does not apply to my country’ were excluded from the analysis. For our exposure variable, respondents were asked to indicate their main source of information on COVID-19. Participants were allowed to provide up to three main sources of information on COVID-19. Potential confounders and predictors of the outcome were included based on an a priori framework informed by the literature (9,29,30) (Figure 1). The following variables were included in our analysis: alternate sources of COVID-19 information (including television, radio, newspapers, family/relatives, employers, and other online/web channels), COVID-19 risk perception, confidence in government COVID-19 response and attitude to COVID-19. Sociodemographic variables like age, sex, level of education and occupation were also included. Where potentially important sociodemographic variables like socioeconomic status were unmeasured, we ensured that we included proxy variables that could potentially account for these variables (Figure 1).

### Data analysis

Simple descriptive analysis was used to summarise the characteristics of study respondents using frequencies and proportions. Unadjusted odds of our outcome given the exposure and covariate were generated using logistic regression models. Thereafter, multivariate logistic regression models were used to estimate the adjusted effect of social media as a main COVID-19 information source on the perceived effectiveness of face masks, using odds ratios (ORs) and 95% confidence intervals (CI). After retaining confounders and predictors identified in the literature (9,29,30), automated backward elimination method based on the Akaike information criterion (AIC) was used to select the final model (31). We also assessed possible effect modifiers and covariate interactions including age, sex and country of residence. No significant interactions were identified, therefore the simpler model was considered as the final model. In terms of model diagnostics, we assessed the model using the area under the operating characteristics curve (AUC) (32), and the Hosmer–Lemeshow goodness-of-fit test (33). Collinearity was assessed using a cut-off for variance inflating factor as < 10.

To assess the robustness of our findings and our multivariate model specification, we conducted a propensity score (PS) matched analysis to balance covariates between the exposure and control groups (34). Covariate balance was assessed using a standardised mean difference (SMD < 0.2) with 1:2 nearest neighbor matching without replacement. All covariates from the main analysis were included in the PS logistic model. All analyses were tested at the 5% significance level and were conducted using R-4.0.2 (35).

### Ethical approval

The survey protocol was approved by the Health Research Development Committee (HRDC) of the Ministry of Health and Wellness, the local institutional review board of Botswana (REF Number HPDME 13/18/1). Informed consent was collected electronically from respondents completing the survey. Participation was voluntary and those who consented were allowed to exit the survey at any time by simply closing the browser page.

## Results

### Study sample characteristics

Among 1988 respondents included in the analysis, 1084 (54.5%) were males, 782 (39.3%) were aged 30–39 years, 1257 (63.2%) resided in urban settings and 522 (26.3%) were from Kenya (Table 1). Further, 1454 (73.1%) felt at risk of COVID-19, while 623 (31.3%) were fearful of COVID-19. A total of 1169 (58.8%) respondents used social media as their main source of information, while 1689 (85.0%) agreed that face masks were effective in reducing the spread of COVID-19.

**Table 1.** Study sample characteristics stratified by social media as main COVID-19 information source, yes or no.

Variables	Overall sample	Main COVID-19 info source: social media – No	Main COVID-19 info source: social media – Yes
	n (%)	n (%)	n (%)
	1988	819	1169
Perceived effectiveness of face masks			
Does not agree	299 (15.0)	139 (17.0)	160 (13.7)
Agree	1689 (85.0)	680 (83.0)	1009 (86.3)
Sex			
Female	846 (42.6)	311 (38.0)	535 (45.8)
Male	1084 (54.5)	472 (57.6)	612 (52.4)
Prefer not to say	58 (2.9)	36 (4.4)	22 (1.9)
Residence			
Peri-urban	421 (21.2)	197 (24.1)	224 (19.2)
Rural	310 (15.6)	142 (17.3)	168 (14.4)
Urban	1257 (63.2)	480 (58.6)	777 (66.5)
Country			
Botswana	489 (24.6)	262 (32.0)	227 (19.4)
Kenya	522 (26.3)	214 (26.1)	308 (26.3)
Malawi	167 (8.4)	74 (9.0)	93 (8.0)
Nigeria	493 (24.8)	146 (17.8)	347 (29.7)
Zambia	179 (9.0)	69 (8.4)	110 (9.4)
Zimbabwe	138 (6.9)	54 (6.6)	84 (7.2)
Confidence in government response			
Very low	150 (7.5)	55 (6.7)	95 (8.1)
Low	264 (13.3)	90 (11.0)	174 (14.9)
Indifferent	435 (21.9)	153 (18.7)	282 (24.1)
High	705 (35.5)	295 (36.0)	410 (35.1)
Very high	434 (21.8)	226 (27.6)	208 (17.8)
Age			
<30 years	565 (28.4)	220 (26.9)	345 (29.5)
30–39 years	782 (39.3)	304 (37.1)	478 (40.9)
40–49 years	481 (24.2)	208 (25.4)	273 (23.4)
50 years and above	160 (8.0)	87 (10.6)	73 (6.2)
Level of education			
Primary/Secondary	179 (9.0)	110 (13.4)	69 (5.9)
Tertiary	1809 (91.0)	709 (86.6)	1100 (94.1)
Occupation			
Employed	1510 (76.0)	610 (74.5)	900 (77.0)
Student	281 (14.1)	109 (13.3)	172 (14.7)
Unemployed/retired	197 (9.9)	100 (12.2)	97 (8.3)
Alternative main COVID-19 info sources^a^			
Television	1257 (63.2)	551 (67.3)	706 (60.4)
Radio	530 (26.7)	286 (34.9)	244 (20.9)
Friends	179 (9.0)	65 (7.9)	114 (9.8)
Family & relatives	107 (5.4)	43 (5.3)	64 (5.5)
Online/Web	577 (29.0)	223 (27.2)	354 (30.3)
Employer	179 (9.0)	84 (10.3)	95 (8.1)
Newspaper	186 (9.4)	96 (11.7)	90 (7.7)
Perceived COVID-19 risk			
Not at risk	534 (26.9)	232 (28.3)	302 (25.8)
At risk	1454 (73.1)	587 (71.7)	867 (74.2)
Attitude to COVID-19			
Calm	339 (17.1)	146 (17.8)	193 (16.5)
Doubt	160 (8.0)	73 (8.9)	87 (7.4)
Fear	623 (31.3)	257 (31.4)	366 (31.3)
Worry	689 (34.7)	286 (34.9)	403 (34.5)
Others	177 (8.9)	57 (7.0)	120 (10.3)

a Multiple response question.

COVID-19: coronavirus disease 2019.

### Association between social media and perceived effectiveness of face masks

Table 2 illustrates the unadjusted and adjusted relationship between social media as main COVID-19 information source and perceived effectiveness of face masks. In unadjusted analysis, respondents who used social media as their main COVID-19 information source, had greater odds of agreeing that face masks were effective compared with those who did not (OR 1.29, 95% CI: 1.01–1.65). This association remained the same when adjusted for age, sex, country, level of education, confidence in government response, attitude towards COVID-19 and alternative main sources of information on COVID-19 (aOR 1.33, 95% CI: 1.01–1.77).

**Table 2.** Estimates from logistic regression assessing the relationship between social media as main COVID-19 information source and perceived effectiveness of face masks.

Variables	Crude relationship	Adjusted relationship
	OR (95% CI)	aOR (95% CI)
Main info source: social media		
No	Reference	Reference
Yes	1.29 (1.01, 1.65)^b^	1.33 (1.01, 1.77)^b^
Sex		
Female	Reference	Reference
Male	0.94 (0.73, 1.21)	1.13 (0.85, 1.51)
Prefer not to say	0.40 (0.22, 0.72)^a^	0.47 (0.24, 0.92)^b^
Residence		
Peri-urban	Reference	
Rural	1.02 (0.67, 1.56)	
Urban	0.93 (0.68, 1.27)	
Country		
Botswana	Reference	Reference
Kenya	2.33 (1.56, 3.47)^a^	4.00 (2.35, 6.81)^a^
Malawi	0.26 (0.17, 0.38)^a^	0.52 (0.31, 0.86)^b^
Nigeria	1.16 (0.82, 1.64)	2.30 (1.44, 3.68)^a^
Zambia	2.76 (1.47, 5.20)^a^	4.49 (2.21, 9.13)^a^
Zimbabwe	1.17 (0.69, 1.99)	2.62 (1.39, 4.96)^a^
Confidence in government response		
Very low	Reference	Reference
Low	1.16 (0.75, 1.80)	1.12 (0.70, 1.80)
Indifferent	2.26 (1.48, 3.45)^a^	2.39 (1.50, 3.79)^a^
High	4.97 (3.23, 7.65)^a^	5.51 (3.41, 8.93)^a^
Very high	4.77 (2.96, 7.67)^a^	6.46 (3.65, 11.41)^a^
Age		
<30 years	Reference	Reference
30–39 years	0.80 (0.58, 1.10)	0.98 (0.66, 1.44)
40–49 years	0.65 (0.46, 0.93)^b^	0.89 (0.58, 1.36)
50 years and above	0.45 (0.29, 0.70)^a^	0.52 (0.30, 0.88)^b^
Level of education		
Primary/Secondary	Reference	Reference
Tertiary	1.48 (1.00, 2.18)^b^	1.51 (0.4, 2.41)
Occupation		
Employed	Reference	
Student	1.59 (1.05, 2.39)^b^	
Unemployed/retired	0.79 (0.54, 1.16)	
Alternative main COVID-19 info^c^ sources		
Television	1.24 (0.97, 1.60)	0.87 (0.66, 1.16)
Radio	1.08 (0.81, 1.43)	1.32 (0.91, 1.91)
Friends	0.80 (0.53, 1.19)	
Family and relatives	0.71 (0.43, 1.16)	
Online/Web	0.72 (0.55, 0.93)^b^	0.73 (0.54, 1.01)
Employer	1.54 (0.94, 2.52)	1.84 (1.08, 3.15)^b^
Newspaper	1.28 (0.81, 2.02)	1.64 (1.00, 2.70)
Perceived COVID-19 risk		
Not at risk	Reference	Reference
At risk	0.97 (0.74, 1.29)	0.93 (0.67, 1.28)
Attitude to COVID-19		
Calm	Reference	Reference
Doubt	0.67 (0.42, 1.09)	0.62 (0.37, 1.06)
Fear	1.30 (0.89, 1.91)	1.21 (0.79, 1.86)
Worry	1.05 (0.73, 1.52)	0.96 (0.64, 1.43)
Others	0.71 (0.44, 1.14)	0.89 (0.53, 1.49)

Adjusted model discrimination and calibration: AUC = 0.77, Archer-Lemeshow (*P* = 0.19).

VIF < 3.

a Significant at *P* < 0.01.

b Significant at *P* < 0.05.

c Reference groups are those who did not indicate using each alternative main source of COVID-19 information.

AUC: area under the operating characteristics curve; COVID-19: coronavirus disease 2019; OR: odds ratio; aOR: adjusted odds ratio; CI: confidence interval; VIF: variance inflating factor.

### PS matching analysis

In sensitivity analysis using PS matching, we achieved considerable improvements in the balance of covariates between exposed and unexposed in the PS matched sample (all SMD < 0.2) compared with the main sample. Table 3 describes the PS-adjusted relationship between using social media as the main source of COVID-19 information and perceived effectiveness of face masks. Findings were similar to those obtained in the main analysis (aOR: 1.44, 95% CI: 1.04, 2.00).

**Table 3.** Sensitivity analysis using PS matching to assess the relationship between social media as main COVID-19 information source and perceived effectiveness of face masks.

Variables	Adjusted association (OR)^a^ (95% CI)
Model: PS matched (1:2 nearest neighbor without replacement)	
Main info source: social media	
No	Reference
Yes	1.44^b,c^ (1.04, 2.00)

a PSs were adjusted for sex, age, country, level of education, confidence in government response, perceived COVID-19 risk, attitude towards COVID-19, first source of information on COVID-19, and alternative main sources of information on COVID-19 including television, radio, friends and family, online/websites, newspapers and employers.

b Propensity score matched estimates not adjusted for sex, age, country, confidence in government response, level of education, perceived COVID-19 risk, attitude towards COVID-19, and alternative main sources of information on COVID-19 including TV, radio, friends and family, online/websites, newspapers and employers.

c Estimate significant at *P* < 0.05.

CI: confidence interval; COVID-19: coronavirus disease 2019; OR: odds ratio; PS: propensity score.

## Discussion

In this study, we found that over half of respondents used social media as their main source of information on COVID-19 and most respondents perceived facemasks to be effective as an NPI for preventing COVID-19. We also found that respondents using social media as their main source of information on COVID 19 had 33% (95% CI: 1–77%) greater odds of perceiving face masks as being effective in preventing COVID-19. This association was significant in the main analysis, and remained significant in sensitivity analysis using PS matching methods to ensure covariate balance between the exposed and control groups.

Findings from this study agree with emerging findings from Africa on the perceived effectiveness of face mask use in preventing COVID-19. For example, a study in Uganda found that over 80% of people perceived face masks to be effective in preventing COVID-19 infections (30). Our findings also support studies suggesting positive associations between information seeking on social media and various aspects of face mask use, including perceived effectiveness. A study in China linked information seeking on social media with perceived effectiveness and compliance with face mask use (36). Another study assessing content from Twitter related to face masks, revealed that clusters of conversations were facilitated by influential accounts run by citizens, politicians and popular culture figures (22). These conversations commonly encouraged the public to wear masks. Further, a study in the United States (US) described personal stories of loss from COVID-19 reported on social media as a motivation to support community use of face masks to prevent COVID-19 (5). Our study provides evidence of the association between the use social media as the main COVID-19 information source and perceived effectiveness of face masks in preventing disease spread, especially in the sub-Saharan context.

Despite the obvious limitations in available evidence, plausible causal explanations for these associations have been proffered. It has been suggested that the personalisation and catchiness of information sharing experiences may explain the association (5). The emotional nature of the messaging in such contexts as exist on social media may also elicit feelings of worry, which have been described as a mediating factor for preventive behaviours such as compliance with face masks (36). However, this mechanism has been disputed, as beliefs about consequences and benefits of face masks may be more important than exposure and belief in misinformation (37).

Our findings support the role of social media as an effective COVID-19 risk communication channel. As successive COVID-19 waves exert their toll on already vulnerable health systems in sub-Saharan Africa, public health interventions leveraging social media may be useful, especially in urban centres where crowding and reliance on subsistent earnings may imply that lockdown measures and stay-at-home orders may not be feasible for extended periods (38). However, health authorities must be aware of the debate about ongoing misinformation via the same channels (13). As has been described, suboptimal regulation, propagation of misinformation based on popularity metrics by social media algorithms and unwitting social media users often spread harmful messages that are often politically motivated (8,17,18,39). Concerted efforts by media, scientific organisations and government institutions are therefore needed to leverage the availability of social media in disseminating important information on the effectiveness of NPIs for COVID-19 including face masks (39), and the benefits of compliance (37).

Future research will be necessary to explore if perceived effectiveness of face masks ultimately result in compliance with mask use. Research will also be necessary to fully understand the mechanisms that result in perceived effectiveness of face mask use in preventing infections with social media use as main source of COVID-19 information. Efforts should also seek to understand the differences in this relationship between various social media platforms. Such information will be useful to inform replicable public health promotion strategies via various social media platforms that are better positioned to influence people’s behaviour to achieve improved health outcomes.

The strengths of our study findings are inherent in the consistency of the observed association in sensitivity analysis using PS matching methods. The association remained significant in both analyses. Moreover, to the best of our knowledge, this is the first study assessing the relationship between social media as a main source of COVID-19 information and perceived effectiveness of face masks in sub-Saharan Africa. This is despite widespread debate about the role of social media misinformation, especially in the context of COVID-19 risk communication. However, our study must also be viewed in light of its limitations. First, our route of participant recruitment implies that the study respondents may not necessarily be representative of the study population of interest. For example, with our online recruitment strategy, respondents included were more likely be those who regularly access online services like social media, and 91% of our sample had tertiary level of education, whereas Nigeria for instance had only 62% adult literacy rates in 2018 (26). However, given that our findings remained consistent in PS analyses where we attempted to account for potential selection bias, we remain confident in our findings. Further, we only recruited 86.3% of our intended sample size and this may have limited the power of our study. We posit that existing fears about government involvement with such types of research may have discouraged participation. Finally, while we considered it expedient to dichotomise our outcome variable for ease of interpretation and applicability to policy discourse, we realise that this may result in loss of statistical information (40).

## Conclusion

In this study of respondents in six sub-Saharan African countries, we found that people who used social media as their main COVID-19 information source were more likely to perceive face mask as effective in preventing COVID-19 spread and this association was statistically significant. With current fears of more deadly waves of infection in the sub-continent, health ministries and agencies may leverage social media to strengthen health promotion messaging on the effectiveness of face masks with a view on promoting widespread mask use.
